# Large Unstained Cells: A Predictive Biomarker for Recurrence and Survival in Resected Gastric Cancer

**DOI:** 10.3390/medicina61020208

**Published:** 2025-01-24

**Authors:** Furkan Ceylan, Ateş Kutay Tenekeci, Burak Bilgin, Mehmet Ali Nahit Şendur, Mutlu Hızal, Fahriye Tuba Köş, Didem Şener Dede

**Affiliations:** 1 Department of Medical Oncology, Ankara Bilkent City Hospital, 06800 Ankara, Turkey; 2 School of Medicine, Hacettepe University, 06230 Ankara, Turkey; 3 Department of Medical Oncology, Ankara Yıldırım Beyazıt University, 06800 Ankara, Turkey

**Keywords:** large unstained cells, gastric cancer, prognostic biomarker, systemic inflammation, survival outcomes

## Abstract

*Background and Objectives:* Despite advances in surgery and perioperative chemotherapy, locally advanced gastric cancer continues to pose significant challenges, creating a pressing need for biomarkers capable of predicting therapeutic efficacy and survival outcomes. This study evaluates the prognostic and predictive significance of large unstained cells (LUCs), a morphologically distinct subset of white blood cells identified in peripheral blood that remain unstained by standard hematological dyes, as potential indicators of immune competence and treatment response. *Materials and Methods:* This retrospective analysis included patients diagnosed with locally advanced gastric cancer (cT2-4, N0-3) at Ankara Bilkent City Hospital between January 2018 and November 2024. Primary endpoints were overall survival (OS) and disease-free survival (DFS), stratified by LUC levels. The secondary endpoint was the association between LUC levels and pathological tumor response. *Results:* A total of 180 patients were analyzed, with a median age of 59 years; a total of 76% were male. The median follow-up period was 16.5 months, during which OS and DFS rates were 82% and 66%, respectively. Most patients were presented with advanced-stage disease, including T3–T4 tumors (91%) and nodal positivity (81%). Stratification by LUC levels revealed significantly shorter DFS (HR: 2.12; 95% CI: 1.12–4.01; *p* = 0.020) and OS (HR: 3.37; 95% CI: 1.26–9.03; *p* = 0.015) in the low-LUC group compared to the high-LUC group. Furthermore, the high-LUC group exhibited a significantly higher tumor shrinkage rate (ypN0: 60% vs. 44%; *p* = 0.020), although tumor regression scores were similar across groups. Advanced tumor stage and lack of pathological response were strongly associated with reduced DFS and OS, while poorly cohesive carcinoma histology emerged as a predictor of inferior OS. *Conclusions:* This study demonstrates that elevated LUC levels are significantly associated with improved DFS and OS, as well as enhanced tumor shrinkage, in patients with locally advanced gastric cancer. These findings show the potential of LUCs as a promising biomarker for prognostication and therapeutic stratification in this population, offering a novel avenue for refining clinical decision-making. Further validation through prospective investigations is warranted.

## 1. Introduction

Gastric cancer is the fifth most common malignancy worldwide and remains the third leading cause of cancer-related mortality [1]. Despite substantial advancements in oncologic care, the prognosis for patients diagnosed with this disease remains dismal, particularly for those with locally advanced or metastatic presentations. Approximately 60–70% of cases are diagnosed at a locally advanced stage, with 5-year survival rates for regional gastric cancer hovering around 35% in the United States [2]. These statistics highlight the significant global burden of gastric cancer and the urgent need for improved therapeutic strategies. Endoscopic biopsy plays a pivotal role in the diagnosis of gastric cancer, providing essential diagnostic and prognostic information. This procedure allows for the assessment of HER2 expression, which serves as a significant prognostic marker, and the evaluation of Microsatellite Stability. Microsatellite status not only holds prognostic value but also guides the selection of neoadjuvant therapies, including immunotherapy, tailored to the molecular characteristics of the tumor [3,4].

Perioperative chemotherapy is the current standard of care for locally advanced gastric cancer, providing superior survival outcomes compared to surgery alone. Landmark trials such as MAGIC [5] and ACCORD-07 [6] established the survival benefit of perioperative ECF (epirubicin, cisplatin, fluorouracil) over surgery alone. More recently, the FLOT4 AIO trial [7] demonstrated that perioperative FLOT (fluorouracil, oxaliplatin, docetaxel) significantly improves overall survival compared to ECF, with median survival durations of 50 months versus 35 months (*p* = 0.012). Additionally, a Japanese study demonstrated that a chemotherapy regimen using S-1 instead of fluorouracil, combined with docetaxel and oxaliplatin, also provides a survival advantage [8]. Consequently, perioperative FLOT is now recommended for younger, fit patients without significant comorbidities, as per current guidelines [9]. However, high recurrence rates persist, signaling the need for innovative biomarkers and therapeutic approaches.

Cancer-associated inflammation is prevalent in the majority of malignancies and is thought to play a pivotal role in promoting tumor progression while potentially diminishing the effectiveness of therapeutic interventions [10,11]. Growing evidence indicates that cancer-related inflammation has a substantial influence on postoperative recovery and long-term prognosis in cancer patients [12,13]. Consequently, inflammation-based biomarkers are emerging as promising predictors of surgical outcomes and overall survival. Prior studies have demonstrated that elevated levels of CD8+ T lymphocytes and memory T cells correlate with improved prognosis, whereas increased neutrophil and platelet counts are associated with poorer survival outcomes [14,15,16,17].

LUCs represent an underexplored component of the systemic inflammatory response. These cells are characterized as large, peroxidase-negative cells that remain unclassified, potentially encompassing activated lymphocytes, peroxidase-negative lymphoblasts, blasts, plasma cells, and hairy cells [18,19]. Elevated LUC levels have been reported in various pathological conditions, including viral infections, melanoma, and leukemias [20]. In chronic lymphocytic leukemia (CLL), a type of hematological malignancy, higher LUC levels have been detected compared to normal levels, and elevated LUC levels have been associated with poor prognosis [20]. In melanoma, elevated LUC levels have been reported; however, the prognostic impact of the LUC-to-lymphocyte ratio has been investigated [21]. Keskin et al. further identified increased LUC levels during viral infections, suggesting that this elevation may signify the presence of abnormal immune cells responding to viral pathogens or tumor cells [22,23]. In locally advanced and metastatic gastric cancer, peripheral blood LUCs may reflect the systemic inflammatory response, with elevated levels potentially correlating with tumor recurrence or progression.

Our study aims to explore the prognostic and predictive value of LUCs in patients with locally advanced gastric cancer. While previous research has predominantly examined the role of LUCs in hematologic malignancies and viral infections, this investigation represents a pioneering effort to assess its clinical significance in solid tumors. To the best of our knowledge, this is the first study to evaluate the absolute LUC count as a prognostic biomarker in locally advanced gastric cancer. By addressing a critical gap in the literature, our findings offer a novel perspective, highlighting LUCs as a potential predictor of recurrence and overall survival. This innovative work sets a foundation for future studies to further elucidate the role of LUCs in oncology.

## 2. Patients and Methods

### 2.1. Patient Selection

This single-center, retrospective study evaluated patients diagnosed with locally advanced gastric cancer at Ankara Bilkent City Hospital between June 2018 and November 2024.

Eligible participants included those with gastric or gastroesophageal junction (GEJ) tumors classified as clinical stage cT2 or higher, exhibiting nodal involvement (cN+) or both, as per the AJCC 8th edition criteria. All included patients received neoadjuvant chemotherapy followed by surgical intervention. Additionally, individuals with peritoneally confined metastases who underwent surgery post-neoadjuvant chemotherapy were incorporated into the analysis.

Comprehensive demographic and clinical data were extracted from medical records, including parameters related to disease-free survival (DFS) and overall survival (OS). Follow-up durations were calculated using the reverse Kaplan–Meier method to account for censoring. The primary endpoints of the study were DFS and OS stratified by LUC levels, while the secondary endpoint examined the relationship between LUC levels and pathological tumor response.

### 2.2. Evaluation of MSI and Tumor Regression Status

In this study, microsatellite status was assessed through immunohistochemical analysis of mismatch repair (MMR) protein expression, including MSH-2, MLH-1, MSH-6, and PMS-2. Deficient mismatch repair (dMMR), indicative of microsatellite instability (MSI), was identified by the loss of expression of either a single MMR protein or a heterodimeric pair within the MMR complex. This approach provided indirect yet reliable evidence of MSI.

Tumor regression was evaluated according to the proportion of viable tumor tissue within the tumor bed, following the classification system described by Becker et al. [24].

Tumor Regression Grades (TRGs) were categorized into four distinct classes:

- TRG1a: Complete pathological response, characterized by the absence of viable tumor cells;
- TRG1b: Major response, defined as less than 10% residual tumor tissue;
- TRG2: Partial regression, denoting 10–50% residual tumor tissue;
- TRG3: Minimal or no regression, with over 50% viable tumor cells and negligible signs of regression within the tumor bed.

This grading system provided a robust framework for quantifying therapeutic response and correlating it with clinical outcomes.

### 2.3. LUC Definition, Analysis, and Sampling Timing

LUCs are a unique subset of white blood cells identified during automated complete blood count (CBC) analysis. These cells, including lymphoblasts, activated lymphocytes, and atypical lymphocytes, remain unclassified due to their atypical characteristics. Blood samples were processed within two hours of collection to ensure reliability, with daily calibration and quality control procedures performed to maintain analyzer accuracy. LUC levels were measured from peripheral blood samples collected at diagnosis less than three days before initiating systemic therapy.

### 2.4. Determination of LUC Levels

LUC levels were assessed using the Siemens ADVIA 2120i hematology analyzer, which employs advanced optical and staining methods to classify blood cells. The process included the following:

Peroxidase Method: Differentiates white blood cells based on peroxidase activity, identifying LUCs as peroxidase-negative cells;

Basophil/Lobularity Method: Uses laser scatter technology to evaluate cell size and internal complexity, categorizing LUCs as large, unclassified cells.

### 2.5. Measurement and Cut-Off Value

LUC levels were reported both as a percentage of the total white blood cell count and an absolute number. Despite performing a ROC analysis, an optimal cut-off value could not be identified. In the absence of an established threshold in the literature, the median LUC level (0.14 K/µL) observed within the study cohort was used to stratify patients into two groups:

Low-LUC group: LUC levels ≤ 0.14 K/µL;

High-LUC group: LUC levels > 0.14 K/µL.

This stratification enabled the evaluation of LUC levels as a potential prognostic biomarker for survival outcomes and disease progression.

### 2.6. Evaluation of Survival

The primary endpoints of this study were DFS and OS, stratified by LUC levels. DFS was defined as the interval from surgery to either disease recurrence or death and OS as the time from diagnosis to either death or the most recent follow-up visit. These endpoints were selected to evaluate the prognostic significance of LUCs in both short- and long-term outcomes.

### 2.7. Statistics

Descriptive statistics were reported as mean ± standard deviation for normally distributed numerical variables and as median with interquartile ranges for variables with non-normal distributions. Categorical variables were presented as counts or percentages. Comparisons between normally distributed numerical variables were performed using Student’s *t*-test, while the Mann–Whitney U test was applied to non-normally distributed data. Proportions of categorical variables were analyzed using the Chi-Square test. DFS and OS were estimated using the Kaplan–Meier method.

Statistical analyses were performed using SPSS version 26.0. A threshold *p*-value of <0.05 was considered indicative of statistical significance. This study received ethical approval from the institutional review board of Ankara Bilkent City Hospital and was conducted in accordance with the Declaration of Helsinki’s guidelines.

## 3. Results

### Patients and Tumor Characteristics

**Figure 1:**
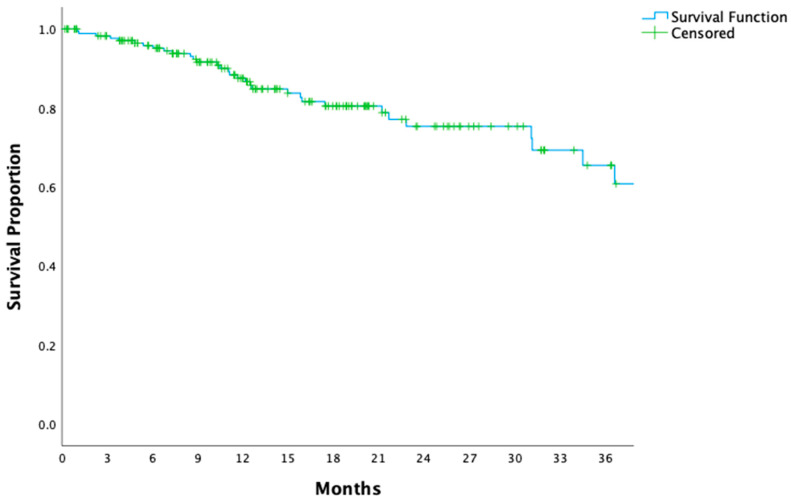
Kaplan–Meier curve for overall survival in patients with locally advanced gastric cancer. The Kaplan–Meier survival curve shows the overall survival (OS) rates for all patients with locally advanced gastric cancer over the follow-up period.

**Figure 2:**
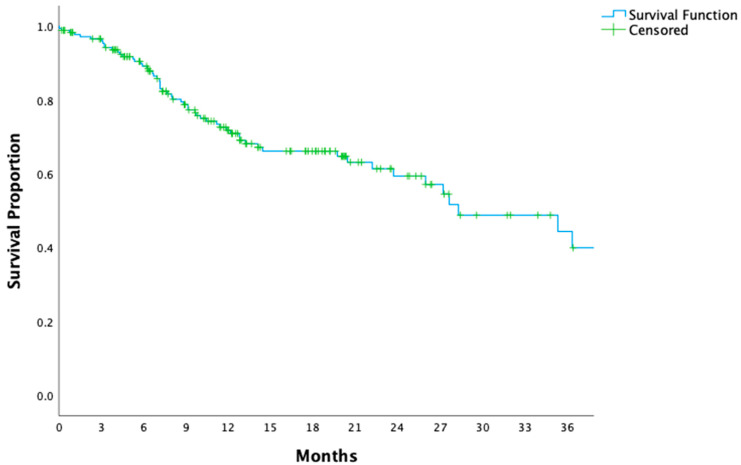
Kaplan–Meier curve for disease-free survival in patients with locally advanced gastric cancer. The Kaplan–Meier survival curve demonstrates disease-free survival (DFS) for the entire cohort of patients with locally advanced gastric cancer.

The study cohort consisted of 180 patients, with a median follow-up duration of 16.5 months. At 16.5 months, OS and DFS rates were 82% and 66%, respectively. The median OS had not been reached, while the median DFS was calculated as 28.3 ± 4.6 months (Figure 1 and Figure 2). Detailed patient, tumor, and surgical characteristics are presented in Table 1.

The median age at diagnosis was 59 years (IQR: 51–66), with 76% of patients being male. ECOG performance status was 0 in 39% (*n* = 70) and 1 in 59% (*n* = 107). Tumor locations were distributed between the gastroesophageal junction (33%) and the stomach (67%). Histologically, adenocarcinoma accounted for 81% of cases, while poorly cohesive carcinoma was observed in 19%. Tumors were moderately or poorly differentiated in 64% of patients. Advanced-stage tumors (T3–T4) were present in 91%, with nodal involvement documented in 81%. The clinical stage distribution was 20% for stage 2, 74% for stage 3, and 6% for stage 4.

Mismatch repair status was determined to be Proficient (pMMR) in 56% of patients, while 12% exhibited Deficient Mismatch Repair (dMMR). In 32% of cases, MMR status could not be assessed. The Charlson Comorbidity Index was <4 in 46% of patients and ≥4 in 54%. The family history of cancer was documented in 28% of cases. The most frequently reported presenting symptoms were dyspepsia (42%) and abdominal pain (35%).

Diagnostic laparoscopy was performed in 77% of the patients. Among the surgical interventions, total gastrectomy was conducted in 71% of cases, while subtotal gastrectomy was performed in 29%. D2 lymph node dissection was achieved in 92% of patients, with R0 resections accomplished in 96% and R1 resections in 4%. Hyperthermic Intraperitoneal Chemotherapy (HIPEC) was administered to 8% of patients. Tumor Regression Grades were distributed as follows: TRG1a in 8%, TRG1b in 13%, TRG2 in 19%, and TRG3 in 37% of cases. Adjuvant chemotherapy was administered to 84% of the cohort.

When stratified by LUC levels, patient characteristics were largely comparable between the low- and high-LUC groups (Table 2). However, poorly cohesive carcinoma histology was significantly less prevalent in the high-LUC group compared to the low-LUC group (7% vs. 23%, *p* = 0.009). Additionally, the ypN0 rate was higher in the high-LUC group (60% vs. 44%, *p* = 0.020), while hematemesis was more frequently observed in the high-LUC group (18% vs. 4%, *p* = 0.005).

**Figure 3:**
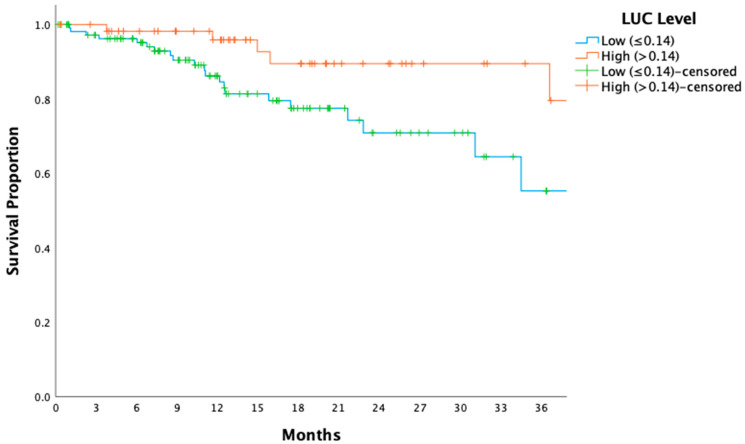
Kaplan–Meier curve for overall survival stratified by large unstained cell levels. The Kaplan–Meier survival curve compares overall survival (OS) between patients with low (≤0.14) and high (>0.14) large unstained cell (LUC) levels. (*p* = 0.015). LUCs: Large Unstained Cells; OS: overall survival.

**Figure 4:**
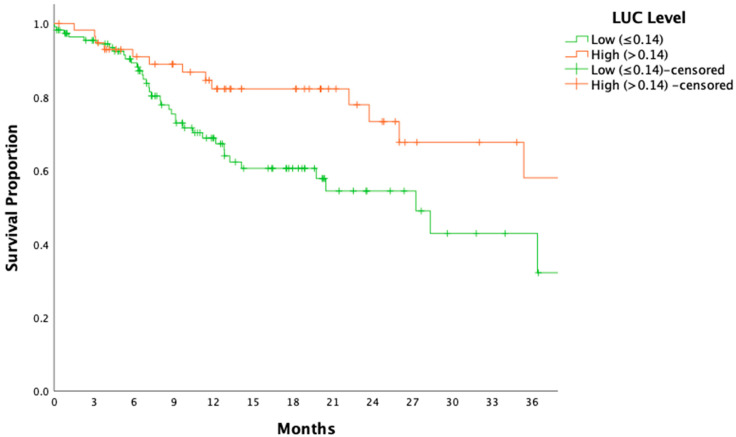
Disease-free survival stratified by LUC levels. The Kaplan–Meier survival curve illustrates disease-free survival (DFS) stratified by large unstained cell (LUC) levels. (*p* = 0.020). LUCs: large unstained cells; DFS: disease-free survival.

Patients with low LUC levels demonstrated significantly shorter DFS compared to those with high LUC levels (HR: 2.12, 95% CI: 1.12–4.01, *p* = 0.020; Figure 3). Similarly, OS was significantly reduced in the low-LUC group (HR: 3.37, 95% CI: 1.26–9.03, *p* = 0.015; Figure 4).

Pathological response rates did not differ significantly between LUC groups (TRG1a: 8% vs. 9%; TRG1b: 12% vs. 16%; TRG2: 16% vs. 26%; TRG3: 39% vs. 33%; *p* = 0.511; Table 2).

Advanced tumor stage was significantly associated with shorter DFS, with hazard ratios of 6.13 (95% CI: 2.05–18.33; *p* = 0.001) for stage 4 versus stage 2 and 2.35 (95% CI: 1.00–5.52; *p* = 0.050) for stage 3 versus stage 2. Similarly, the absence of pathological tumor response (TRG3 vs. TRG1a–2) was strongly correlated with reduced DFS (HR: 6.25, 95% CI: 2.63–14.29; *p* < 0.001). Conversely, no significant differences in DFS were observed based on age (<65 vs. ≥65; HR: 0.88, 95% CI: 0.51–1.52; *p* = 0.645), sex (male vs. female; HR: 1.01, 95% CI: 0.53–1.91; *p* = 0.986), tumor location (GEJ vs. gastric; HR: 1.23, 95% CI: 0.72–2.10; *p* = 0.442), type of surgery (subtotal vs. total gastrectomy; HR: 0.72, 95% CI: 0.39–1.31; *p* = 0.279), or ECOG performance status (ECOG 0 vs. 1–2; HR: 0.69, 95% CI: 0.39–1.18; *p* = 0.169; Table 3).

Similarly, advanced tumor stage was a significant predictor of shorter OS, with hazard ratios of 8.71 (95% CI: 2.15–35.26; *p* = 0.002) for stage 4 versus stage 2 and 2.78 (95% CI: 1.35–5.56; *p* = 0.005) for stage 3 versus stage 2. The presence of poorly cohesive carcinoma was also associated with reduced OS (HR: 2.78, 95% CI: 1.35–5.56; *p* = 0.005), as was the lack of pathological tumor response (TRG3 vs. TRG1a–2; HR: 7.69, 95% CI: 2.33–25.00; *p* = 0.001). In contrast, no significant differences in OS were observed based on age (<65 vs. ≥65; HR: 0.93, 95% CI: 0.45–1.91; *p* = 0.847), sex (male vs. female; HR: 0.51, 95% CI: 0.25–1.05; *p* = 0.067), tumor location (GEJ vs. gastric; HR: 0.84, 95% CI: 0.40–1.76; *p* = 0.642), type of surgery (subtotal vs. total gastrectomy; HR: 0.51, 95% CI: 0.22–1.17; *p* = 0.111), or ECOG performance status (ECOG 0 vs. 1–2; HR: 0.81, 95% CI: 0.40–1.62; *p* = 0.543; Table 4).

## 4. Discussion

This study revealed a significant association between elevated LUC levels and improved clinical outcomes, including longer DFS and OS, in patients with locally advanced gastric cancer. As shown in Figure 3 and Figure 4, patients with higher LUC levels exhibited superior DFS and OS compared to those with lower levels, highlighting the potential role of LUCs as a robust prognostic biomarker. Additionally, tumor shrinkage rates were higher in the high-LUC group, a finding consistent with the ypN0 rates observed in Table 2 (60% vs. 44%, *p* = 0.020). These results suggest that elevated LUC levels reflect a critical link between immune system activation and tumor dynamics, potentially contributing to enhanced tumor control and delayed disease progression.

Higher LUC levels may indicate a more robust immune response, characterized by heightened activity of cytotoxic lymphocytes or other immune effector cells capable of suppressing tumor proliferation. This enhanced immune competence likely facilitates greater tumor control, as evidenced by the higher tumor shrinkage observed in the high-LUC group (Table 2). Conversely, advanced tumor stage and absence of pathological response were strongly associated with poorer outcomes (Table 3 and Table 4), correlating with significantly reduced DFS and OS. The identification of poorly cohesive carcinoma as an independent negative prognostic factor for DFS further emphasizes the complexity of tumor biology, as this histological subtype is associated with aggressive behavior, therapeutic resistance, and poorer survival rates.

In the management of locally advanced gastric cancer, the integration of perioperative chemotherapy and surgery remains the cornerstone of treatment. Among patients with optimal ECOG performance status and minimal comorbidities, perioperative FLOT has emerged as the most effective regimen, yielding superior survival rates in both clinical trials and real-world applications [7]. However, as demonstrated in Figure 1 and Figure 2, recurrence and progression rates remain substantial, showing the persistent challenge of achieving durable long-term disease control. This highlights the need for innovative biomarkers like LUCs to refine prognostic assessments and guide personalized therapeutic strategies.

Inflammation-based scoring systems, which incorporate peripheral blood markers, have been widely employed to predict prognosis in locally advanced gastric cancer. Established markers such as the NLR (neutrophil-to-lymphocyte ratio) and the PLR (platelet-to-lymphocyte ratio) have demonstrated utility in stratifying recurrence risk and survival outcomes by assessing the roles of neutrophils, lymphocytes, and platelets [25,26]. Recent efforts to enhance the predictive accuracy of these tools have incorporated additional parameters, such as albumin levels, reflecting the interplay between systemic inflammation and nutritional status [27].

Building on this foundation, our study explored whether LUCs, a parameter derived from automated complete blood count analysis, could serve as a reliable biomarker for predicting recurrence, survival outcomes, and pathological response. The findings revealed that elevated LUC levels were associated with significantly improved DFS and OS, suggesting that LUCs may reflect a critical component of systemic immune competence (Figure 3 and Figure 4). Previously, LUCs have been characterized as a population of peroxidase-negative cells, including prolymphocytes, lymphoblasts, blasts, and plasma cells. Lymphocytes, which play pivotal roles in cellular and humoral antitumor immune responses, are central to mediating cytotoxic cell death, suppressing tumor proliferation, and inhibiting metastatic migration [28]. The elevated LUC levels observed in this study likely encompass lymphoid precursors, such as lymphoblasts and prolymphocytes, indicative of heightened immune activity. This enhanced immune response may underlie the improved clinical outcomes seen in patients with higher LUC levels, reflecting a more robust and effective antitumor immune mechanism.

The study by Fortes et. al. [21] examined the percentage of LUCs and the LUC-to-lymphocyte ratio as inflammatory markers with potential prognostic implications in melanoma. However, their analysis found no significant association between these markers and overall survival. In contrast, another study investigating B-CLL reported a positive correlation between LUC levels and lymphocyte counts [20]. While similar associations might exist across other malignancies, including solid tumors, the prognostic utility of the LUC-to-lymphocyte ratio remains complex and inconsistent, warranting further investigation

Departing from the approach employed by Fortes et al. [21], this study uniquely focuses on the absolute LUC count rather than ratios or percentages, providing a distinct perspective. To the best of our knowledge, this is the first study to evaluate the prognostic and predictive significance of absolute LUC levels in gastric carcinoma. This novel approach addresses a previously unexplored dimension of cancer biomarker research, offering insights into the role of LUCs in disease progression and survival outcomes.

Notably, this study did not identify a direct relationship between LUC levels and pathological response in patients with locally advanced gastric cancer. However, it is essential to recognize that tumor regression assessments predominantly target the primary tumor, often overlooking the pathological status of lymph nodes. Intriguingly, our analysis revealed a higher rate of metastatic lymph node clearance following neoadjuvant therapy in patients with elevated LUC levels, suggesting a link between LUCs and enhanced tumor shrinkage. This finding highlights the potential of LUCs as an independent prognostic biomarker for locally advanced gastric cancer, particularly in reflecting systemic treatment responsiveness.

In locally advanced gastric cancer, several prognostic indicators are well established, including advanced age, sarcopenia, larger tumor size, deeper invasion, poor histological differentiation, the presence of metastatic lymph nodes, limited lymph node dissection (D1), and inadequate pathological response to therapy [29,30,31,32]. Additionally, super-extended lymphadenectomy, as demonstrated in the study by Marrelli et al., has been identified as a key factor in improving survival outcomes, highlighting the importance of thorough lymph node clearance in surgical management [33]. For patients with peritoneal metastatic gastric cancer, innovative approaches such as Pressurized Intraperitoneal Aerosol Chemotherapy (PIPAC) have shown promise in improving survival and potentially enabling conversion surgery in selected cases. However, further prospective studies are required to validate their efficacy and define their optimal application in clinical practice [34,35]. These factors collectively show the intricate interplay between tumor biology and host-related factors in determining clinical outcomes. In our study, these risk factors were evenly distributed between the high- and low-LUC groups, ensuring balanced comparisons and minimizing potential confounding effects. This balance allowed for a more accurate evaluation of the prognostic significance of LUC levels in locally advanced gastric cancer.

Consistent with the existing literature, our findings highlighted that a lack of pathological tumor response (TRG 3) and advanced tumor stage were significantly associated with poorer DFS and OS, showing their critical role in determining prognosis. Advanced-stage disease reflects a greater tumor burden and more extensive dissemination, while suboptimal pathological responses indicate reduced sensitivity to systemic therapy, both of which are pivotal determinants of treatment failure and disease progression. These results emphasize the importance of achieving effective tumor regression and early-stage detection to improve survival outcomes in patients with locally advanced gastric cancer.

*Helicobacter pylori* (*H. pylori*) infection is a well-established risk factor for the development of gastric cancer and is known to contribute to chronic gastritis, peptic ulcer disease, and gastric carcinogenesis [36,37]. Its role in cancer progression is mediated by various virulence factors that target cellular pathways regulating immune responses, inflammation, and tumorigenesis. Given its critical impact, the relationship between H. pylori and systemic immune markers, such as LUCs, warrants further investigation.

Although our study provides novel insights into the prognostic significance of LUC levels in patients with locally advanced gastric cancer, we did not evaluate the frequency of H. pylori infection at the time of diagnosis. This omission represents a limitation of our work, as H. pylori infection may influence systemic inflammatory markers and, consequently, LUC levels. Future research should explore the potential interplay between H. pylori infection and LUC, as such studies could deepen our understanding of immune modulation in gastric cancer and identify additional prognostic or predictive biomarkers.

Overall, this study shows the multifaceted nature of prognostic factors in locally advanced gastric cancer, highlighting the critical role of both systemic immune biomarkers and traditional clinicopathological parameters in shaping clinical outcomes. By demonstrating the prognostic significance of LUC levels, this research contributes to the growing body of evidence advocating for the integration of immune-based biomarkers into routine clinical practice. These findings not only offer a novel perspective on the biological mechanisms underlying tumor progression but also pave the way for future studies aimed at validating LUCs as a reliable tool for risk stratification and personalized treatment planning. As recurrence rates remain alarmingly high despite advancements in multimodal therapy, continued exploration of innovative biomarkers such as LUCs is imperative for refining therapeutic strategies and improving long-term survival in gastric cancer patients.

## 5. Conclusions

This study establishes the prognostic significance of LUCs, a marker of systemic immune activity, in locally advanced gastric cancer. Elevated LUC levels were significantly associated with improved DFS and OS, along with higher tumor shrinkage rates, positioning them as a potential biomarker for predicting recurrence and survival outcomes. By focusing on absolute LUC counts, this research explores a previously uncharted area of gastric cancer biology and offers new insights into the relationship between systemic inflammation and clinical outcomes.

These findings contribute to a deeper understanding of the role of immune response in tumor dynamics and point to the potential integration of LUCs into clinical practice. As recurrence rates remain a persistent challenge despite advancements in treatment strategies, the ability of LUCs to reflect immune competence and treatment responsiveness makes them a promising tool for risk stratification and personalized therapeutic approaches.

Future prospective studies are needed to validate these results across broader patient cohorts and investigate the underlying mechanisms linking LUCs to immune modulation and tumor control. Establishing LUCs as a reliable biomarker may enhance prognostic accuracy, guide therapeutic decisions, and ultimately lead to improved survival outcomes for patients with gastric cancer.

## Figures and Tables

**Table 1:** Baseline clinical and tumor characteristics of the patient cohort. This table presents the demographic, clinical, and tumor-specific characteristics of the study population. Statistical significance was defined as *p* < 0.05. IQR: interquartile range; ECOG: Eastern Cooperative Oncology Group; GEJ: gastroesophageal junction; pMMR: Proficient Mismatch Repair; dMMR: Deficient Mismatch Repair; HIPEC: Hyperthermic Intraperitoneal Chemotherapy; TRG: Tumor Regression Grade; ypT/ypN/ypM: Pathological Staging After Neoadjuvant Therapy; D: Dissection Staging; R: resection staging; LUCs: large unstained cells.

		Whole (*n* = 180)
Age, Median, IQR		59 (51–66)
Gender, Male		137 (76%)
ECOG	0	70 (39%)
	1	107 (59%)
	2	3 (2%)
Localization	GEJ	60 (33%)
	Gastric	120 (67%)
Histology	Adenocarcinoma	146 (81%)
	Poorly cohesive	34 (19%)
Grading according to the WHO	Unknown	47 (26%)
	Grade 1	18 (10%)
	Grade 2	42 (23%)
	Grade 3	73 (41%)
Stage T	T1	0 (0%)
	T2	12 (7%)
	T3	124 (69%)
	T4	44 (24%)
Stage N	N0	34 (19%)
	N1	86 (48%)
	N2	45 (25%)
	N3	15 (8%)
Stage	2	36 (20%)
	3	133 (74%)
	4	11 (6%)
Microsatellite Status	Unknown	58 (32%)
	pMMR	101 (56%)
	dMMR	21 (12%)
Charlson Comorbidity Index	<4	82 (46%)
	≥4	98 (54%)
Family History		51 (28%)
Smoking		84 (47%)
Alcohol		9 (5%)
Chief Complaint	Dyspepsia	74 (42%)
	Abdominal pain	62 (35%)
	Weight Loss	11 (6%)
	Hematemesis/Melena	14 (8%)
	Dysphagia	28 (16%)
Diagnostic Laparoscopy		139 (77%)
Surgery	Total gastrectomy	127 (71%)
	Subtotal gastrectomy	53 (29%)
Dissection	D1	15 (8%)
	D2	165 (92%)
Resection	R0	172 (96%)
	R1	8 (4%)
Use of HIPEC		15 (8%)
Number of Lymph Nodes Excised, Median, IQR		26 (20–34)
ypT	ypT0	18 (10%)
	ypT1	25 (14%)
	ypT2	17 (9%)
	ypT3	90 (50%)
	ypT4	30 (17%)
ypN	ypN0	86 (48%)
	ypN1	35 (19%)
	ypN2	29 (16%)
	ypN3	30 (17%)
Metastases	M1	11 (6%)
ypM	ypM1	9 (5%)
Tumor Regression Score	TRG X	43 (23%)
	TRG 1a	14 (8%)
	TRG 1b	23 (13%)
	TRG 2	34 (19%)
	TRG 3	66 (37%)
Adjuvant Chemotherapy		143 (84%)
Relapse		58 (32%)
Death		34 (19%)

**Table 2:** Patient and tumor characteristics according to the LUC level. This table compares baseline clinical, demographic, and tumor-specific features between patients with low (≤0.14) and high (>0.14) large unstained cell (LUC) levels. Statistical significance was defined as *p* < 0.05. IQR: interquartile range; ECOG: Eastern Cooperative Oncology Group; GEJ: gastroesophageal junction; pMMR: Proficient Mismatch Repair; dMMR: Deficient Mismatch Repair; HIPEC: Hyperthermic Intraperitoneal Chemotherapy; TRG: Tumor Regression Grade; ypT/ypN/ypM: Pathological Staging After Neoadjuvant Therapy; D: Dissection Staging; R: resection staging; LUC: large unstained cells.

		LUCs ≤ 0.14 (*n* = 113)	LUCs > 0.14 (*n* = 58)	*p*
Age, Median, IQR		59 (49–65)	61 (53–68)	0.113
Gender, Male		83 (74%)	48 (83%)	0.173
ECOG	0	44 (39%)	24 (41%)	0.280
	1	66 (58%)	34 (59%)	
	2	3 (3%)	0 (0%)	
Localization	GEJ	39 (35%)	19 (33%)	0.819
	Gastric	74 (65%)	39 (67%)	
Histology	Adenocarcinoma	87 (77%)	54 (93%)	0.009
	Poorly cohesive	26 (23%)	4 (7%)	
Grading according to the WHO	Unknown	30 (26%)	16 (27%)	0.162
	Grade 1	12 (11%)	5 (9%)	
	Grade 2	21 (19%)	19 (33%)	
	Grade 3	50 (44%)	18 (31%)	
Stage T	T1	0 (0%)	0 (0%)	0.068
	T2	4 (4%)	7 (12%)	
	T3	82 (72%)	35 (60%)	
	T4	27 (24%)	16 (28%)	
Stage N	N0	22 (20%)	12 (21%)	0.138
	N1	59 (52%)	24 (41%)	
	N2	26 (23%)	13 (22%)	
	N3	6 (5%)	9 (16%)	
Stage	2	22 (19%)	13 (22%)	0.194
	3	81 (72%)	44 (76%)	
	4	10 (9%)	1 (2%)	
Microsatellite Status	Unknown	37 (33%)	21 (36%)	0.201
	pMMR	64 (56%)	28 (48%)	
	dMMR	12 (11%)	9 (16%)	
Charlson Comorbidity Index	<4	57 (50%)	21 (36%)	0.077
	≥4	56 (50%)	37 (64%)	
Family History		33 (29%)	15 (26%)	0.645
Smoking		50 (44%)	29 (50%)	0.475
Alcohol		3 (3%)	6 (10%)	0.063
Chief Complaint	Dyspepsia	49 (44%)	22 (39%)	0.521
	Abdominal pain	35 (31%)	22 (39%)	0.340
	Weight loss	7 (6%)	4 (7%)	1.000
	Hematemesis/Melena	4 (4%)	10 (18%)	0.005
	Dysphagia	21 (19%)	6 (10%)	0.162
Diagnostic Laparoscopy		93 (82%)	45 (78%)	0.460
Surgery	Total gastrectomy	82 (73%)	38 (66%)	0.340
	Subtotal gastrectomy	31 (27%)	20 (34%)	
Dissection	D1	8 (7%)	5 (9%)	0.764
	D2	105 (93%)	53 (91%)	
Resection	R0	109 (97%)	56 (97%)	1.000
	R1	4 (3%)	2 (3%)	
Use of HIPEC		13 (12%)	2 (3%)	0.078
Number of Lymph Nodes Excised, Median, IQR		25 (19–34)	28 (23–38)	
ypT	ypT0	12 (11%)	6 (10%)	0.658
	ypT1	14 (12%)	11 (19%)	
	ypT2	10 (9%)	7 (12%)	
	ypT3	57 (50%)	27 (47%)	
	ypT4	20 (18%)	7 (12%)	
ypN	ypN0	50 (44%)	35 (60%)	0.020
	ypN1	30 (27%)	4 (7%)	
	ypN2	18 (16%)	9 (16%)	
	ypN3	15 (13%)	10 (17%)	
Metastases	M1	10 (9%)	1 (2%)	0.101
ypM	ypM1	6 (5%)	3 (5%)	1.000
Tumor Regression Score (TRG)	TRG X	28 (25%)	10 (17%)	0.511
	TRG 1a	9 (8%)	5 (9%)	
	TRG 1b	14 (12%)	9 (16%)	
	TRG 2	18 (16%)	15 (26%)	
	TRG 3	44 (39%)	19 (33%)	
Adjuvant Chemotherapy		88 (85%)	50 (88%)	0.590
Relapse		38 (34%)	13 (24%)	0.129
Death		22 (20%)	5 (9%)	0.065

**Table 3:** Univariate analysis for disease-free survival. This table presents the univariate hazard ratios (HRs) and 95% confidence intervals (CIs) for disease-free survival (DFS) based on clinical, pathological, and treatment-related variables. Statistical significance was defined as *p* < 0.05. HR: hazard ratio; CI: confidence interval; DFS: disease-free survival; ECOG: Eastern Cooperative Oncology Group; TRG: Tumor Regression Grade; LUCs: large unstained cells; GEJ: gastroesophageal junction; CCI: Charlson Comorbidity Index; MSS: Microsatellite Stability; D: Dissection Staging.

	Univariate Analyses		
	HR	95% CI	*p*
Age < 65 vs. ≥65	0.88	0.51–1.52	0.645
Male to Female	1.01	0.53–1.91	0.986
Presence of Family History	1.04	0.59–1.86	0.883
High CCI (>4 vs. ≤4)	1.09	0.65–1.83	0.742
GEJ vs. gastric	1.23	0.72–2.10	0.442
Adenocarcinoma vs. Poorly Cohesive Cancer	1.54	0.85–2.77	0.154
Well and Intermediate vs. Poorly Differentiated	1.05	0.59–1.87	0.881
MSS or not	0.80	0.35–1.84	0.606
Stage 3 vs. 2	2.35	1.00–5.52	0.050
Stage 4 vs. 2	6.13	2.05–18.33	0.001
Subtotal vs. Total Gastrectomy	0.72	0.39–1.31	0.279
D2 vs. D1 Dissection	0.64	0.29–1.42	0.274
TRG 1a-1b-2 vs. 3	0.16	0.07–0.38	<0.001
ECOG 0 vs. 1–2	0.68	0.39–1.18	0.169
LUC Low (≤0.14) vs. LUC High (>0.14)	2.12	1.12–4.01	0.020

**Table 4:** Univariate analysis for overall survival. This table presents the univariate hazard ratios (HRs) and 95% confidence intervals (CIs) for overall survival (OS) based on clinical, pathological, and treatment-related variables. Statistical significance was defined as *p* < 0.05.HR: hazard ratio; CI: confidence interval; DFS: disease-free survival; ECOG: Eastern Cooperative Oncology Group; TRG: Tumor Regression Grade; LUCs: large unstained cells; GEJ: gastroesophageal junction; CCI: Charlson Comorbidity Index; MSS: Microsatellite Stability; D: Dissection Staging.

	Univariate Analyses		
	HR	95% CI	*p*
Age <65 vs. ≥65	0.93	0.45–1.91	0.847
Male to Female	0.51	0.25–1.05	0.067
Presence of Family History	1.07	0.50–2.29	0.870
High CCI (>4 vs. ≤4)	1.08	0.55–2.12	0.832
GEJ vs. Gastric	0.84	0.40–1.76	0.642
Adenocarcinoma vs. Poorly Cohesive Cancer	0.36	0.18–0.74	0.005
Well and Intermediate vs. Poorly Differentiated	0.78	0.37–1.66	0.518
MSS or not	1.14	0.44–2.94	0.790
Stage 3 vs. 2	2.27	0.69–7.53	0.180
Stage 4 vs. 2	8.71	2.15–35.26	0.002
Subtotal vs. Total Gastrectomy	0.51	0.22–1.17	0.111
D2 vs. D1 Dissection	0.49	0.19–1.26	0.138
TRG 1a-1b-2 vs. 3	0.13	0.04–0.43	0.001
ECOG 0 vs. 1–2	0.81	0.40–1.62	0.543
LUC Low (≤0.14) vs. LUC High (>0.14)	3.37	1.26–9.03	0.015

## Data Availability

The data supporting the findings of this study are available from the corresponding author upon reasonable request. In adherence to ethical standards and institutional policies, only fully anonymized datasets will be shared to protect patient confidentiality. Data access will be granted exclusively for legitimate academic and non-commercial research purposes, following a formal request and subject to approval in line with institutional and ethical regulations.
